# Exogenous detection of ^13^C-glucose metabolism in tumor and diet-induced obesity models

**DOI:** 10.3389/fphys.2022.1023614

**Published:** 2022-10-05

**Authors:** Amandine Verlande, Sung Kook Chun, Wei A. Song, Daniela Oettler, Harm J. Knot, Selma Masri

**Affiliations:** ^1^ Department of Biological Chemistry, University of California, Irvine, Irvine, CA, United States; ^2^ TSE Systems Inc., Chesterfield, MO, United States

**Keywords:** tumor metabolism, diet-induced obesity (DIO), insulin resistance, glucose oxidation and detection, circadian clock

## Abstract

Metabolic rewiring is a hallmark feature prevalent in cancer cells as well as insulin resistance (IR) associated with diet-induced obesity (DIO). For instance, tumor metabolism shifts towards an enhanced glycolytic state even under aerobic conditions. In contrast, DIO triggers lipid-induced IR by impairing insulin signaling and reducing insulin-stimulated glucose uptake. Based on physiological differences in systemic metabolism, we used a breath analysis approach to discriminate between different pathological states using glucose oxidation as a readout. We assessed glucose utilization in lung cancer-induced cachexia and DIO mouse models using a U-^13^C glucose tracer and stable isotope sensors integrated into an indirect calorimetry system. Our data showed increased ^13^CO_2_ expired by tumor-bearing (TB) mice and a reduction in exhaled ^13^CO_2_ in the DIO model. Taken together, our findings illustrate high glucose uptake and consumption in TB animals and decreased glucose uptake and oxidation in obese mice with an IR phenotype. Our work has important translational implications for the utility of stable isotopes in breath-based detection of glucose homeostasis in models of lung cancer progression and DIO.

## Introduction

The circadian clock system coordinates organismal behavior and physiology within a 24-hour time period. This biological pacemaker governs rhythms in sleep/wake activity, feeding/fasting cycles, endocrine regulation, and metabolism. The circadian clock is entrained by environmental cues such as light, feeding, exercise, and temperature (Takahashi, 2017; Guan and Lazar, 2021). Deregulation of the circadian clock has been linked with several pathologies, including endocrine disruption, IR, and cancer (Shi et al., 2013; Masri and Sassone-Corsi, 2018; Stenvers et al., 2019; Verlande and Masri, 2019). Meal timing and dietary composition have been shown to rewire the circadian clock and systemic metabolism (Kohsaka et al., 2007; Hatori et al., 2012; Eckel-Mahan et al., 2013). Additionally, genetic clock disruption in mice impacts insulin secretion (Perelis et al., 2015), hepatic glucose production and export (Rudic et al., 2004; Lamia et al., 2008), postprandial glucose disposal in skeletal muscle (Dyar et al., 2014), and results in obesity and type 2 diabetes (Turek et al., 2005; Marcheva et al., 2010; Paschos et al., 2012; Perelis et al., 2015). In the context of cancer, clock-controlled uptake, transport, and metabolism of glucose is disrupted (Altman et al., 2015; Papagiannakopoulos et al., 2016). Importantly, tumors also distally rewire metabolism to alter systemic insulin and glucose signaling (Masri et al., 2016; Hojo et al., 2017; Verlande et al., 2021).

Metabolic rewiring is one of the hallmarks of cancer that promotes tumor proliferation in low nutrient and oxygen conditions (Hanahan, 2022). Metabolic reprogramming includes the Warburg effect or aerobic glycolysis, and the recycling of lactate, amino acids and ammonia to support cancer cell growth and progression (Vander Heiden et al., 2009; Hensley et al., 2016; Spinelli et al., 2017). Interestingly, lactate has been found to be the primary fuel source of the mitochondrial tricarboxylic acid (TCA) cycle in human lung tumors (Hensley et al., 2016; Faubert et al., 2017; Hui et al., 2017) and in many other tissues (Hui et al., 2017). The Warburg effect has been demonstrated in a vast majority of tumors, and clinical applications have been leveraged for imaging, such as 2-deoxy-2-[fluorine-18]fluoro-D-glucose Positron Emission Tomography (^18^F-FDG-PET) to detect glucose uptake in tumors (Vaquero and Kinahan, 2015). Also, increased glucose oxidation through glycolysis and the TCA cycle have been demonstrated in tumor tissues compared to surrounding non-cancerous tissues using ^13^C-glucose carbon tracing (Fan et al., 2009; Hensley et al., 2016). Taken together, studies from humans and mice show that increased glucose and lactate oxidation in the mitochondria is characteristic of altered metabolism of lung tumors and other tumor types.

Glucose homeostasis is also disrupted in metabolic syndrome, obesity and type 2 diabetes (Rosen and Spiegelman, 2006; Yang et al., 2018). Consumption of high fat and high sugar diets is one of the major causes of DIO (Malik et al., 2013), and is attributed to IR that is characterized by elevated blood glucose and triglyceride levels (Saltiel and Kahn, 2001; Eckel et al., 2005). Insulin facilitates glucose uptake and inhibits hepatic gluconeogenesis and glycogenolysis. In addition, insulin activates *de novo* lipogenesis and reduces lipolysis in adipocytes, thereby lowering circulating lipid content (Saltiel and Kahn, 2001; Czech, 2017). IR attenuates these processes and results in hyperglycemia and hyperlipidemia that is associated with heightened inflammation (Arkan et al., 2005), endoplasmic reticulum stress (Ozcan et al., 2004), and ectopic lipid deposition in tissues (Krssak et al., 1999; Fabbrini et al., 2009). Also, IR impacts mitochondrial dynamics and plasticity and the ability to switch from fatty acid to glucose oxidation in skeletal muscle (Ukropcova et al., 2007; Koves et al., 2008). Collectively, DIO-induced IR disrupts systemic glucose homeostasis.

**FIGURE 1:**
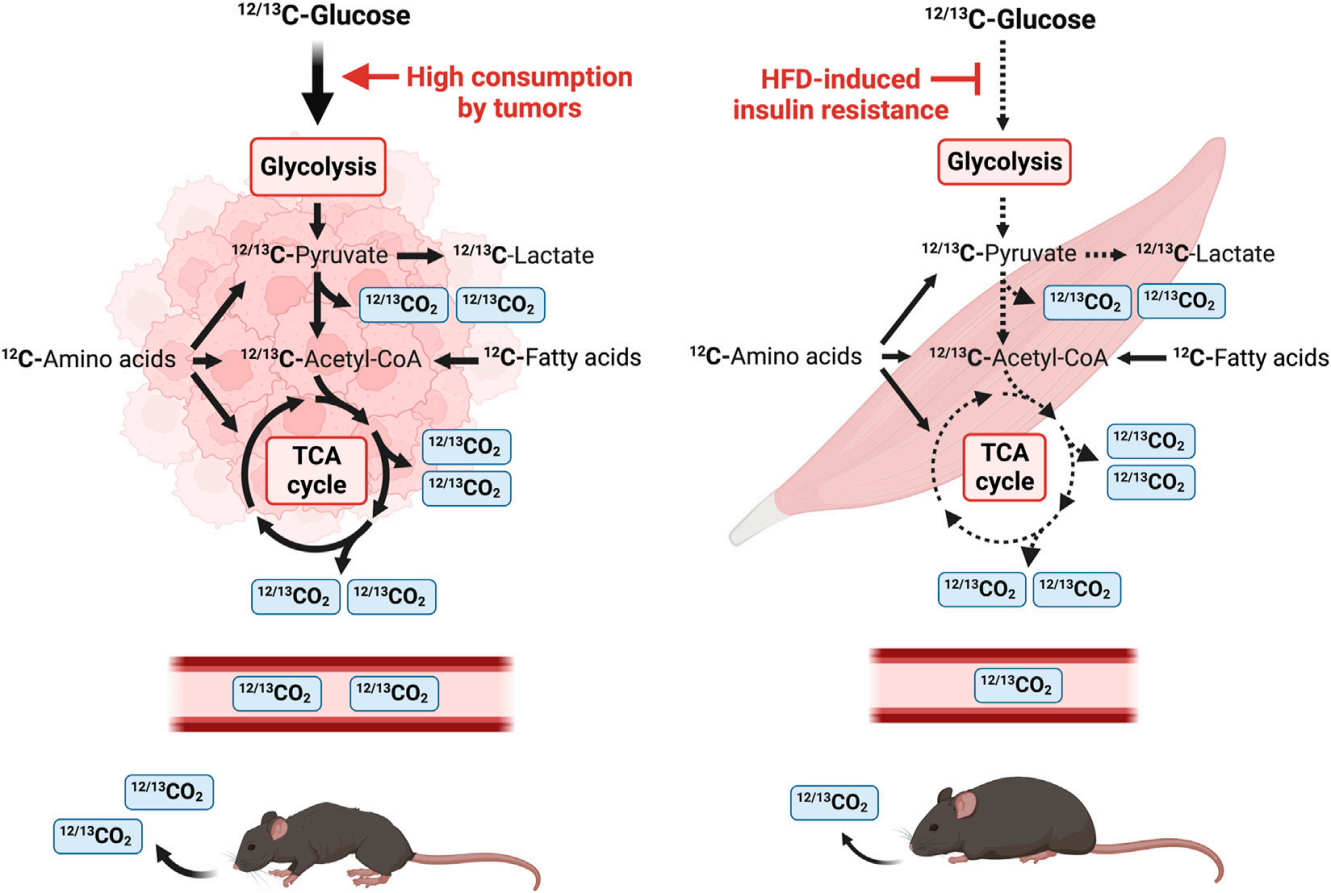
Schematic diagram of a stable isotope breath test study in mice using U-^13^C glucose. U-^13^C glucose tracer is introduced by intraperitoneal injection and the amount of ^13^CO_2_ and total CO_2_ is measured by a nondispersive infrared spectrometer. Tumor tissue consumes a mix of ^12^C and ^13^C glucose that is metabolized through glycolysis. ^13^C-pyruvate, the end-product of glycolysis, is either converted to ^13^C-lactate or enters the TCA cycle in the mitochondria. One CO_2_ is released from pyruvate oxidation to acetyl-CoA and two CO_2_ molecules are released per round of the TCA cycle. Tumors heavily consume glucose and tumor-bearing mice release a larger amount of ^12^CO_2_ and ^13^CO_2_ in the air. In contrast, HFD-induced IR leads to a reduction in systemic glucose uptake and utilization, and this is reflected in a reduction of expired ^12^CO_2_ and ^13^CO_2_.

In this study, we illustrate that metabolic alterations of glucose utilization can be leveraged for real-time detection of lung tumor progression and DIO. We utilize a ^13^C-glucose tracer, that upon injection, can be detected in real-time in exhaled breath to quantify glucose uptake and consumption using stable isotope sensors in combination with indirect calorimetry (Figure 1). We confirmed the increased glucose uptake and consumption in a lung cancer cachexia model using an exogenous breath-based approach with a ^13^C-glucose tracer and stable isotope sensors. Using this same technology, we also illustrate that glucose uptake and oxidation is reduced in a model of DIO, which accurately reflects an IR phenotype. Overall, the utility of stable isotopes is becoming prevalent for metabolic fate mapping (Buescher et al., 2015; Jang et al., 2018; Lopes et al., 2021; Diehl et al., 2022; Pachnis et al., 2022) and clinical diagnostics (Modak, 2007; Braden, 2009), and we demonstrate that use of ^13^C-glucose faithfully recapitulates metabolic deregulation in models of lung tumor progression and DIO.

## Results

### Rationale for the ^13^C breath assay

The breakdown of carbohydrates, lipids, and proteins into monosaccharides, fatty acids and amino acids generates ATP and provides cellular energy. These monomers are processed to acetyl-CoA and other metabolites of the TCA cycle (Figure 1). Monosaccharides such as glucose are further processed during glycolysis to produce two molecules of pyruvate, ATP and CO_2_ per molecule of glucose (Figure 1). Pyruvate is subsequently metabolized to lactate or shuttled into the mitochondrial matrix where it is converted to acetyl-CoA or oxaloacetate in the TCA cycle. Decarboxylation occurs twice in the TCA cycle, and four molecules of CO_2_ are produced per molecule of glucose (Figure 1). Therefore, six molecules of CO_2_ can be produced per molecule of glucose which is transported by red blood cells to be exhaled by the lungs. Use of a stable isotope tracer such as ^13^C-glucose allows for accurate quantification of ^13^CO_2_, relative to total CO_2_, which is detected by stable isotope sensors (Figure 1) (Fernández-Calleja et al., 2019; Seichter et al., 2021). Tumors are well-known to be avid metabolizers of glucose, and many metabolites are found at higher levels in lung cancer tissues versus surrounding non-cancerous tissues (Fan et al., 2009), indicating activated glycolysis and enhanced TCA cycle in tumors. As CO_2_ is released from the TCA cycle, these data suggest that TB mice release more CO_2_ derived from glucose than healthy animals. In contrast, DIO induces IR and results in a reduction of glucose uptake in skeletal muscle and a decrease of released CO_2_ (Figure 1).

### Metabolic alterations in a mouse model of lung cancer-associated cachexia

**FIGURE 2:**
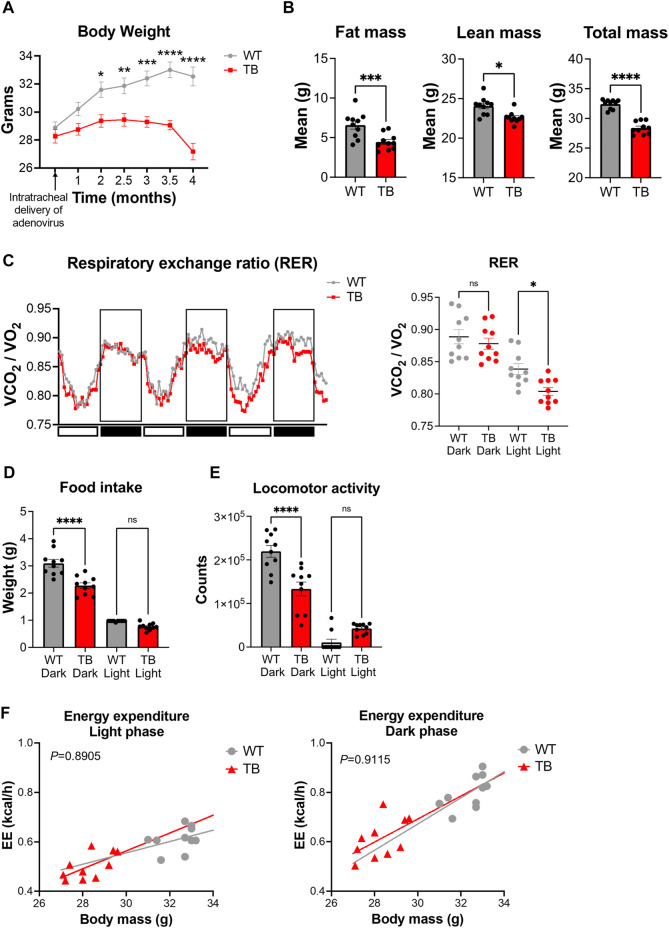
Metabolic phenotyping of a mouse model of lung cancer-associated cachexia. **(A)** Body weight over the course of 4 months post-intratracheal delivery of the adenovirus FlpO or Cre recombinase. Data represent the mean ± SEM with *p* value cutoff indicated as *p < 0.05, **p < 0.01 ***p < 0.001, and ****p < 0.0001 as determined by two-way ANOVA. **(B)** Fat, lean, and total mass measurements at 4 months post-delivery of adenovirus (*n* = 10 mice per genotype). Data represent the mean ± SEM with *p* value cutoff indicated as *p < 0.05, ***p < 0.001, and ****p < 0.0001 as determined by Student’s *t*-test. **(C)** (Left) RER (VCO_2_/VO_2_) in WT and TB mice over 3 days. (Right) Quantification of the mean RER in WT and TB mice over 4 days (*n* = 10 mice). Data represent the mean ± SEM with *p* value cutoff indicated as *p < 0.05 as determined by one-way ANOVA. **(D)** Food intake of WT and TB mice over 4 days (*n* = 10 mice). Data represent the mean ± SEM with *p* value cutoff indicated as ****p < 0.0001 as determined by one-way ANOVA. **(E)** Locomotor activity in WT and TB mice over 4 days (*n* = 10 mice). Data represent the mean ± SEM with *p* value cutoff indicated as ****p < 0.0001 as determined by one-way ANOVA. **(F)** Linear regression plots of TEE are the average heat production (H3 in kcal/h) plotted to body mass during the dark and light phases. Formula of the regression plot from the light phase for WT mice is y = 0.023 × −0.1346 and TB mice is y = 0.03616–0.5214. For the dark phase, formula of the regression plot for WT mice is y = 0.05309 × −0.9206 and TB mice is y = 0.04664–0.7068. *p* values for group differences in slopes are 0.6193 for light phase and 0.8599 for dark phase. ns, not significant.

The *Kras* ^*LSL-G12D/+*^ *;p53* ^*fl/fl*^ genetically engineered mouse model (GEMM) of lung cancer mirrors human non–small cell lung cancer and exhibits cancer-associated cachexia (CAC) (Jackson et al., 2001; Jackson et al., 2005; Papagiannakopoulos et al., 2016). Administration of the viral Cre recombinase *via* the trachea induces the genetic rearrangement of the Lox-stop-Lox cassette to activate oncogenic Kras and delete the tumor suppressor *p53*. Lung tumors developed uniformly with 100% penetrance and tumors can distinctively be observed two to 3 months post-infection (Masri et al., 2016; Verlande et al., 2021). WT mice had an average weight of 32.54 g versus 27.18 g for TB mice at 4 months post-infection (Figure 2A), indicating that lung TB mice exhibited a significant weight loss compared to WT mice. Consistent with the development of cachexia, TB mice displayed a loss of adipose tissue and skeletal muscle as determined by fat mass and lean mass measurement using EchoMRI (magnetic resonance imaging) (Figure 2B), which is consistent with our previous findings (Masri et al., 2016; Verlande et al., 2021). Metabolic phenotyping analysis using indirect calorimetry was performed to better characterize this GEMM. The respiratory exchange ratio (RER) is the ratio between the amount of CO_2_ that is produced to the amount of O_2_ that is consumed. RER oscillates with a daily pattern, reaching the lowest point during the light/resting phase and the highest point during the dark/active phase (Figure 2C). The amount of CO_2_ that is produced in the TCA cycle and the amount of O_2_ that is consumed during oxidative phosphorylation varies depending on the nutrients (monosaccharides, fatty acids, amino acids) that are used for substrate utilization. If glucose is used as a substrate, RER will be approximately 1, as six molecules of O_2_ are required to produce six molecules of CO_2_ for glucose oxidation. Most glucose molecules are derived from diet and oxidation occurs during the dark/active phase in mice. Compared to carbohydrates, fatty acids require more O_2_ molecules for oxidation and therefore the RER value will be less than 1. Fatty acid oxidation occurs during the light/resting phase when carbohydrates are not available from the diet. Interestingly, we observed a significant difference in the RER during the light phase, where TB mice exhibited a lower RER (Figure 2C). These data indicate that TB mice utilize fatty acids as a fuel source during the resting phase, more than the WT mice.

Next, we looked at the food consumption and locomotor activity, two parameters that contribute to energy homeostasis. We observed a significant decrease in food intake (Figure 2D) and locomotor activity (Figure 2E) in TB mice during the dark/active phase, even though we did not observe differences in RER values between WT and TB mice (Figure 2C). We then looked at the total energy expenditure (TEE) relative to body weight using a regression-based approach (Kaiyala and Schwartz, 2011; Tschöp et al., 2011). TEE can be divided into activity energy expenditure (AEE) and resting energy expenditure (REE) (Hills et al., 2014). TB mice displayed a similar AEE during the dark/active phase and comparable REE during the light/resting cycle compared to WT despite the presence of lung tumors (Figure 2F). However, skeletal muscle is the main tissue contributing to TEE (Tschöp et al., 2011; Goncalves et al., 2018; Queiroz et al., 2022) and TB mice exhibited decreased lean mass and locomotor activity (Figures 2D,E). Taken together, these data suggest that the decreased supply of dietary energy is compensated by a reduction in energy consumed for movement as AEE and REE were similar between both genotypes.

### Metabolic perturbation in a mouse model of diet-induced obesity

**FIGURE 3:**
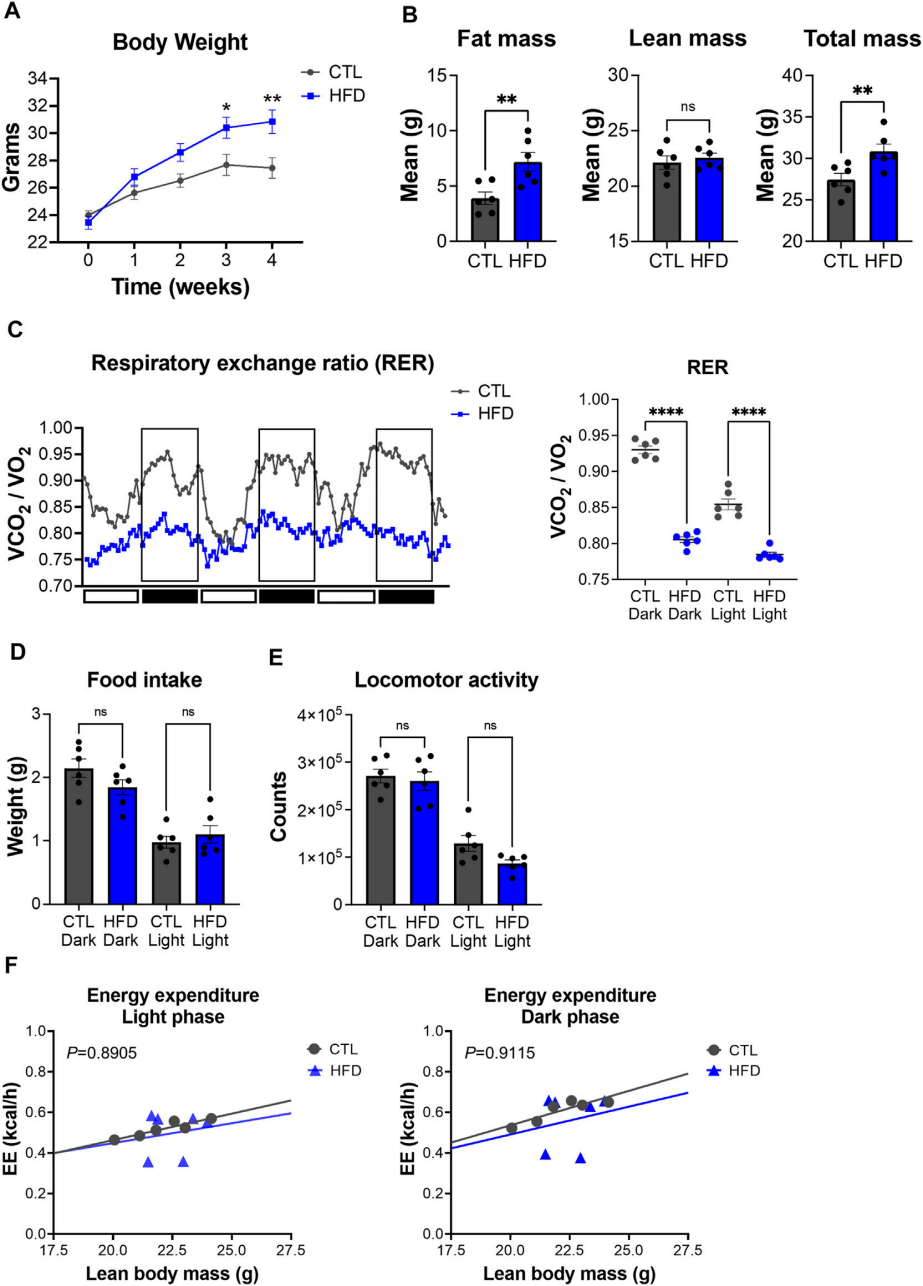
Metabolic phenotyping of a mouse model of DIO. **(A)** Body weight over the course of 4 weeks with feeding of low-fat control diet (CTL) or HFD. Data represent the mean ± SEM with *p* value cutoff indicated as *p < 0.05 and **p < 0.01 as determined by two-way ANOVA. **(B)** Fat, lean, and total mass measurements at 4 weeks after feeding of CTL or HFD (*n* = 6 mice per group). Data represent the mean ± SEM with *p* value cutoff indicated as **p < 0.01 determined by Student’s *t*-test. **(C)** (Left) RER (VCO_2_/VO_2_) in CTL and HFD fed mice over 3 days. (Right) Quantification of the mean RER in CTL and HFD fed mice over 4 days (*n* = 6 mice). Data represent the mean ± SEM with *p* value cutoff indicated as ****p < 0.0001 as determined by one-way ANOVA. **(D)** Food intake in CTL and HFD fed mice over 4 days (*n* = 6). Data represent the mean ± SEM and statistical significance was determined by one-way ANOVA. **(E)** Locomotor activity in CTL and HFD fed mice over 4 days (*n* = 6). Data represent the mean ± SEM and statistical significance was determined by one-way ANOVA. **(F)** Linear regression plots of TEE are the average heat production (H3 in kcal/h) plotted to lean body mass during the dark and light phases. Formula of the regression plot from the light phase for CTL mice is y = 0.02609 × −0.05851 and HFD mice is y = 0.0196 + 0.05654. For dark phase, formula of the regression plot for CTL mice is y = 0.03397 × −0.1431 and HFD mice is y = 0.02740 × −0.05685. *p* values for group differences in slopes 0.8905 for light phase and 0.9115 for dark phase. ns, not significant.

To characterize metabolic deregulation in DIO, male C57BL/6J mice were fed with control low-fat (CTL, 10% kcal from fat) or high-fat diet (HFD, 45% kcal from fat) for 4 weeks. Body weight changes were measured weekly and showed that HFD fed mice gained significantly more body weight than CTL diet fed mice, starting from the third week of feeding (Figure 3A). After 4 weeks of feeding, CTL diet fed mice had an average body weight of 27.45 g versus 30.85 g for the HFD fed group (Figure 3A). To further validate the DIO phenotype, we performed whole-body composition analysis of these mice by EchoMRI. Average fat mass of HFD fed mice was 7.19 g versus 3.90 g for the CTL fed group (Figure 3B). Conversely, average lean mass in HFD fed mice was not significantly different from CTL diet fed mice (Figure 3B), suggesting that body weight gain in HFD fed mice is a result of fat accumulation. These results show that 4 weeks of HFD feeding promotes fat accumulation and weight gain, without a significant impact on lean mass, which is consistent with previous studies (Fisher-Wellman et al., 2016).

To further dissect metabolic dysfunction, we performed indirect calorimetry with our DIO mouse model. While CTL diet fed mice showed rhythmicity of RER over the day/night cycle, HFD fed mice display a significant reduction of RER, which was accompanied with a loss of daily rhythmicity (Figure 3C). These data suggest that HFD fed mice have a higher contribution of fat oxidation as an energy source rather than carbohydrates, which is a hallmark feature of DIO (Eckel et al., 2005). Unlike RER, HFD feeding induced no significant changes in locomotor activity and food intake in both light and dark phases (Figures 3D,E). TEE changes relative to lean body mass did not differ between CTL and HFD fed mice (Figure 3F). These results suggest that metabolic alterations found in our DIO mouse model are not derived from changes in food intake or physical activity, but from fat used as a substrate for oxidation under HFD feeding. Overall, our data demonstrate that HFD fed mice harbor metabolic deficits that model DIO and IR.

### Real-time U-^13^C glucose oxidation in two pathophysiological mouse models

**FIGURE 4:**
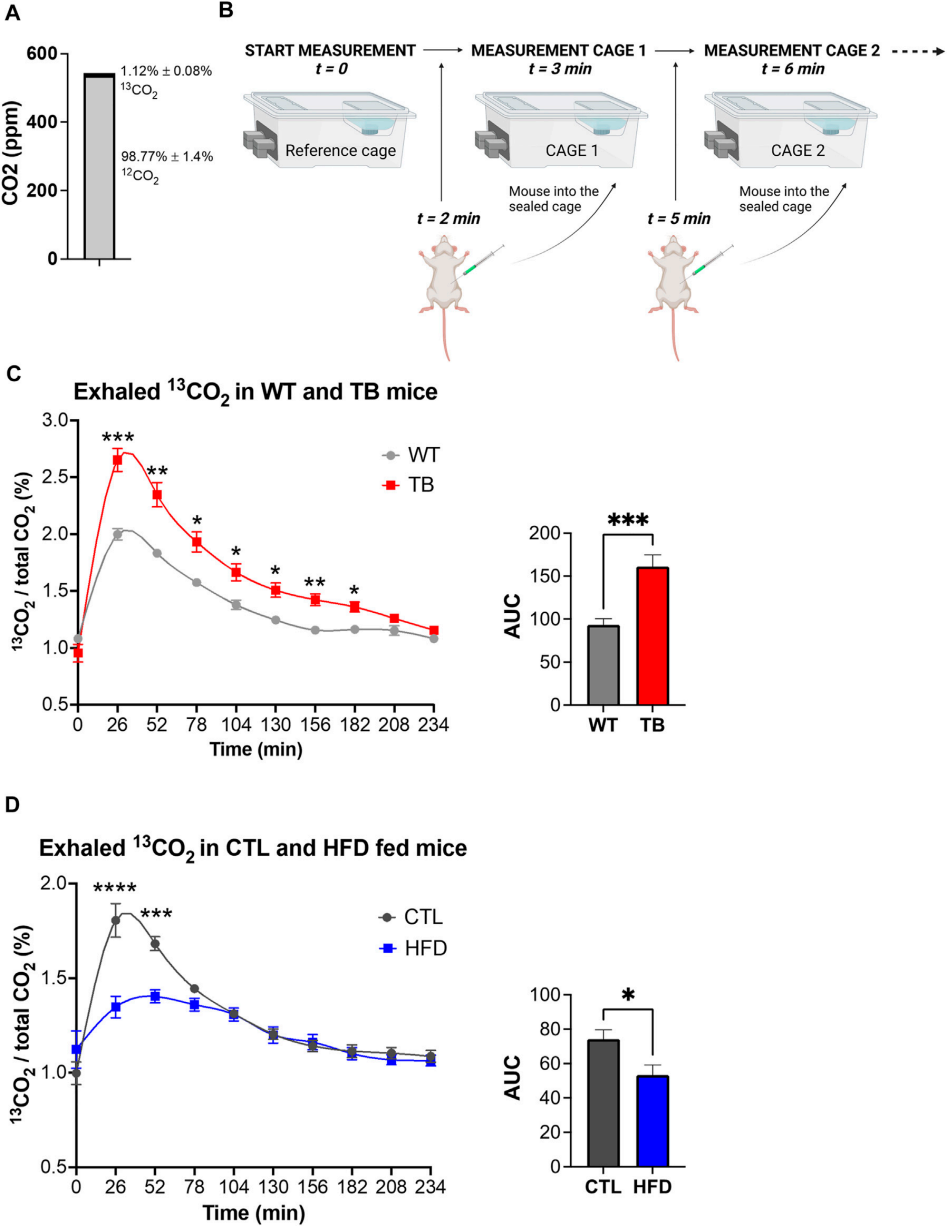
Glucose oxidation in two pathophysiological mouse models. **(A)** Environmental levels of ^13^CO_2_ and ^12^CO_2_ in the metabolic cages. **(B)** Experimental design depicting the reference cage and two of the experimental cages. **(C)** Measurements of exhaled ^13^CO_2_ normalized to total CO_2_ and area under the curve (AUC) in WT and TB mice (*n* = 10 mice per genotype). Data represent the mean ± SEM with *p* value cutoff indicated as *p < 0.05, **p < 0.01, and ***p < 0.001 as determined by two-way ANOVA and Student’s *t*-test. **(D)** Measurements of exhaled ^13^CO_2_ normalized to total CO_2_ and area under the curve (AUC) in CTL and HFD fed mice (*n* = 6 mice per diet type). Data represent the mean ± SEM with *p* value cutoff indicated as *p < 0.05 and ***p < 0.001, and ****p < 0.0001 as determined by two-way ANOVA and Student’s *t*-test.

Considering the phenotypic changes that were observed in both mouse models, systemic glucose metabolism was assessed by measuring exhaled ^13^CO_2_ and total CO_2_ using a ^13^C-glucose tracer and stable isotope sensors. Environmental levels of ^12^C and ^13^C isotopes in the sealed cages were determined prior to the start of the experiment and were 98.77% ± 1.4% and 1.12% ± 0.08% for ^12^CO_2_ and ^13^CO_2_, respectively (Figure 4A). This is in accordance with the natural abundance of ^13^CO_2_ of 1.109%. The experimental design is depicted in Figure 4B. Each mouse received a mixture of 0.2 g/kg of ^12^C-glucose and 0.1 g/kg of ^13^C-glucose, and real-time detection of glucose uptake and consumption was determined. We observed a significant increase in oxidation of ^13^C-glucose in TB mice compared to WT mice (Figure 4C). The level of ^13^CO_2_ in the air reached 2.75% in TB mice compared to 2% in WT after 26 min. Glucose oxidation was significantly elevated in TB mice up to 182 min. These data demonstrate that lung TB mice uptake and oxidize more glucose than WT littermates.

Using a similar approach, we measured ^13^C-glucose oxidation in the DIO mouse model. Each mouse received a mixture of 0.2 g/kg of ^12^C-glucose and 0.1 g/kg of ^13^C-glucose based on their lean body mass. The ratio of expired ^13^CO_2_ to ^12^CO_2_ was measured and a significant reduction was found in HFD fed mice compared to CTL (Figure 4D), suggesting a decrease in glucose oxidation. The maximum level of ^13^CO_2_ in the air reached 1.5% in HFD fed mice versus 2% in WT after 26 min. Taken together, TB mice oxidize more glucose than WT littermates, while HFD fed mice uptake and oxidize less glucose than CTL fed mice. These results demonstrate that the uptake and oxidation of glucose vary in healthy and disease states and that stable isotope sensors accurately quantify glucose oxidation in lung cancer and DIO mouse models.

## Discussion

In this study, we performed a metabolic analysis of the phenotypic changes and glucose utilization in a model of lung cancer-associated cachexia and DIO. We demonstrated that energy metabolism is altered in both pathophysiological mouse models. The lung cancer-associated cachexia GEMM exhibited a reduction in food intake, lean and fat mass, suggesting increased global catabolism (Baracos et al., 2018). Active protein and lipid turnover are observed in patients with cachexia and whole-body proteolysis and lipolysis rates increase by 40% and 50%, respectively (Hall and Baracos, 2008). Also, the rate of fatty acid release is greater in cachexic versus non-cachexic animals (Beck and Tisdale, 2004) and we observed an increased fat oxidation during the resting phase in TB mice. Insulin and glucagon regulate lipolysis and we previously showed that serum glucagon is elevated (Verlande et al., 2021) and insulin signaling is dampened in late-stage lung TB mice (Masri et al., 2016), preventing insulin from exerting its anti-lipolytic effect. In addition, proinflammatory cytokines stimulate lipolysis and fat oxidation in humans (van Hall et al., 2003), and increased tumor-secreted IL-6 is characteristic in animal models of cancer cachexia (Strassmann et al., 1992; Das et al., 2011; Petruzzelli et al., 2014; Masri et al., 2016), as well as in cachectic cancer patients (Staal-van den Brekel et al., 1995; Weidle et al., 2010). We did not observe differences in activity or resting energy expenditure despite the presence of lung tumors. However, skeletal muscle is a primary determinant of TEE (Speakman, 2013; Goncalves et al., 2018; Queiroz et al., 2022) and TB mice exhibited decreased lean mass and locomotor activity. In contrast, early-onset obese mice did not display changes in lean mass or locomotor activity but exhibited increased body weight and fat mass. Body mass is strongly correlated with diabetes and IR and elevated plasma levels of non-esterified fatty acids have been shown to account for up to 50% of IR, which impairs glucose utilization in peripheral tissues (Boden, 2002).

Glucose is considered one the most important circulating energy precursors and we show that its use is oppositely regulated in these pathophysiological models. Our findings highlight that lung TB animals exhibited enhanced glucose uptake, oxidation and mitochondrial function, and this is in accordance with previous studies (Fan et al., 2009; Hensley et al., 2016). It has been reported that increased mitochondrial function is mainly due to elevated pyruvate carboxylation in lung tumors (Fan et al., 2009; Sellers et al., 2015). In addition, oncogenic signaling enhances expression of glucose transporters and glycolytic enzymes in cancer cells, which results in increased glucose uptake and subsequent biomass production (Vander Heiden and DeBerardinis, 2017). Additionally, lung tumors impinge on hepatic metabolism at a distance by rewiring insulin signaling and promoting *de novo* glucose production (Masri et al., 2016; Verlande et al., 2021), thereby disrupting the circadian rhythmicity of plasma glucose.

In the context of DIO, our results showed that IR reduced glucose uptake, oxidation, and mitochondrial function, and resulted in increased fat oxidation. Skeletal muscle plays a critical role in glucose homeostasis and is responsible for uptake of 70%–90% of the glucose from the blood in the postprandial state (DeFronzo et al., 1981; Baron et al., 1988). Previous studies demonstrated that IR in skeletal muscle can be attributed to impaired insulin signaling, reduced insulin-stimulated glucose uptake and transport, and reduced glucose transporter type 4 (GLUT4) translocation (Cline et al., 1994; Ciaraldi et al., 1995; Roden et al., 1996; Dresner et al., 1999; Griffin et al., 1999; Leto and Saltiel, 2012). It has been shown that muscle clock disruption reduces protein levels of GLUT4 and impairs insulin-stimulated glucose uptake (Dyar et al., 2014) and that high-fat diet alters clock-controlled genes governing fuel utilization (Kohsaka et al., 2007; Eckel-Mahan et al., 2013). Additionally, it has been reported that obesity-related IR in skeletal muscle is defined by hyperactivated mitochondrial β-oxidation, impairing the transition from fatty acid to carbohydrate oxidation during the fasted to fed state (An et al., 2004; Koves et al., 2005; Koves et al., 2008). Indeed, glucose and glucose-6-phosphate are reduced in skeletal muscle of diabetic patients (Cline et al., 1994; Cline et al., 1999) and during a lipid infusion, implying that glucose uptake is the rate-limiting step in DIO.

^13^C-glucose breath tests have been reported as an alternative method to measure insulin sensitivity to screen pre-diabetic and diabetic individuals, in contrast to the invasive hyperinsulinemic-euglycemic clamp (Mizrahi et al., 2010; Hussain et al., 2014). Exhaled breath test analysis may also be a promising approach for cancer detection (Dharmawardana et al., 2020; Meng et al., 2021; Kwon et al., 2022). To date, several breath tests have been successfully applied in the clinic for the assessment of gastroenterological symptoms and diseases. ^13^C-urea breath tests have been used to detect *Helicobacter pylori* infection in the stomach, where labeled urea is metabolized to produce ^13^CO_2,_ which is transported to the lung to be exhaled (Alzoubi et al., 2020). Additionally, ^13^C-gastric breath tests have been used to document gastric emptying time (Keller et al., 2018). ^13^C-liver function breath tests can measure the elimination of a substance which is exclusively cleared by the liver (Dietrich et al., 2016). These tests have been used in patients with liver fibrosis and cirrhosis (Giannini and Savarino, 2013; Afolabi et al., 2018), and hepatocellular carcinoma (Palmieri et al., 2009). Finally, ^13^C-pancreatic function breath tests have been used to evaluate the exocrine function of the pancreas which is often disrupted in chronic pancreatitis and pancreatic cancer (Keller and Layer, 2005). While the ^13^C-urea breath is highly standardized and well-established, the ^13^C-gastric, liver and pancreatic breath tests are not widely used due to a lack of standardization. In this study, we provide evidence that altered systemic metabolism can be leveraged using ^13^C-glucose as a method to track lung cancer progression and hallmarks of DIO.

## Materials and methods

### Animal housing and experimental procedures

Male 7-month-old C57BL/6J *Kras* ^*LSL-G12D/+*^ *;p53* ^*fl/fl*^ mice have been previously described (Jackson et al., 2001; Johnson et al., 2001; Jackson et al., 2005; DuPage et al., 2009). Littermates were housed in a standard 12-hour light/dark paradigm and fed *ad libitum* with standard vivarium chow (TEKLAD Envigo 2020X Global Soy Protein-free Extruded Rodent Diet). For viral infection, ad5-CMV: FlpO or ad5-CMV: Cre (University of Iowa, Viral Vector Core) were used at a titer of 3 × 10^7^ plaque-forming units and administered by intratracheal delivery. The intratracheal instillation method has been described previously (Jackson et al., 2001; Jackson et al., 2005). Equivalent titer of viral FlpO recombinase (ad5-CMV: FlpO) was delivered to *p53* ^*fl/fl*^ littermates of the same C57BL/6J background as a control that does not induce recombination to maintain wild-type expression of Kras and *p53* (Masri et al., 2016; Verlande et al., 2021). At 3.75 month after viral infection, metabolic phenotyping of the mice was performed by indirect calorimetry. Male 8-week-old C57BL/6J mice were purchased from The Jackson Laboratory (#00064, Bar Harbor, ME). Mice were divided randomly into two groups and fed *ad libitum* with high-fat (45% calories from fat, D12451, Research Diet) or low-fat control (CTL) diet (10% calories from fat, D12450H, Research Diet) for 4 weeks. Body weight changes were measured every week. Metabolic phenotyping of the mice was performed by indirect calorimetry at 4 weeks after dietary challenge. All experiments were performed in accordance with the Institutional Animal Care and Use Committee (IACUC) guidelines at the University of California, Irvine.

### Indirect calorimetry and echo magnetic resonance imaging

Oxygen consumption (ml/h), carbon dioxide release (ml/h), respiratory exchange ratio (RER), locomotor activity (counts), and food intake (grams) were monitored for individually housed mice using the Phenomaster metabolic cages (TSE Systems Inc., Chesterfield, MO). The climate chamber was set to 21°C, 50% humidity with a 12:12 light-dark cycle as the home-cage environment. Animals were entrained for 2 days in the metabolic cages before the start of each experiment to allow for environmental acclimation. Data were collected at 40-minute intervals and each cage was recorded for 3.25 min before time point collection. Body composition was measured using EchoMRI^™^ Whole Body Composition Analyzer (Houston, TX, United States) which provides whole body fat and lean mass measurements.

### *In vivo* sensing of ^13^CO_2_ and total CO_2_

Mice were acclimated for 2 days before the experiment. Mice were morning fasted for 6 h before and during the stable isotope experiment. Environmental levels of ^13^CO_2_ and total CO_2_ in the sealed cages were calibrated in order to have a percent of ± 1.1% ^13^CO_2,_ as the natural abundance of ^13^C. Measurements were started at t = 0 min, the first mouse was intraperitoneally injected at t = 2 min with a solution of 0.2 g/kg of ^12^C glucose mixed with 0.1 g/kg of U-^13^C glucose, and placed into the first sealed cage. The second mouse was IP injected at t = 5 min when the first cage was being measured. Each cage was measured for 3 min. Exhaled ^13^CO_2_ for each cage was normalized to total CO_2_ abundance to quantify changes in systemic glucose utilization, without accounting for differences in total respiration. Overall, changes of exhaled ^13^CO_2_ levels over time were analyzed by area under the curve (AUC) analysis after subtraction of the area below 1% which is considered baseline.

## Data Availability

The raw data supporting the conclusions of this article will be made available by the authors, without undue reservation.
